# Consumption of multiple micronutrients or small-quantity lipid-based nutrient supplements containing iodine at the recommended dose during pregnancy, compared with iron and folic acid, does not affect women’s urinary iodine concentration in rural Malawi: a secondary outcome analysis of the iLiNS DYAD trial

**DOI:** 10.1017/S1368980020003250

**Published:** 2020-10-15

**Authors:** Seth Adu-Afarwuah, Charles D Arnold, Kenneth Maleta, Per Ashorn, Ulla Ashorn, Josh M Jorgensen, Yue-Mei Fan, Minyanga Nkhoma, Jaden Bendabenda, Andrew Matchado, Kathryn G Dewey

**Affiliations:** 1 Department of Nutrition and Food Science, University of Ghana, Legon, Accra, Ghana; 2 Institute for Global Nutrition and Department of Nutrition, University of California, Davis, CA, USA; 3 University of Malawi College of Medicine, School of Public Health and Family Medicine, Department of Public Health, Blantyre, Malawi; 4 Center for Child Health Research, Tampere University Faculty of Medicine and Health Technology and Tampere University Hospital, Tampere, Finland

**Keywords:** iLiNS DYAD-Malawi, Small-quantity lipid-based nutrient supplements, Multiple micronutrients, Pregnant women, Iodine intakes, Urinary iodine concentration

## Abstract

**Objectives:**

Inadequate iodine intake during pregnancy increases the risk of neonatal morbidity and mortality. We aimed to evaluate whether prenatal supplements containing iodine affect urinary iodine concentrations (UIC) of pregnant women in Malawi.

**Design:**

A randomised controlled trial. Pregnant women (*n* 1391) were assigned to consume 60 mg/d Fe and 400 µg/d folic acid (IFA) or 18 vitamins and minerals including 250 µg/d iodine (MMN) or 20 g/d small-quantity lipid-based nutrient supplements (SQ-LNS) with similar nutrient contents as MMN group, plus macronutrients (LNS) until childbirth. In a sub-study (*n* 317), we evaluated group geometric mean urinary iodine concentration (UIC) (µg/L) at 36 weeks of gestation controlling for baseline UIC and compared median (baseline) and geometric mean (36 weeks) UIC with WHO cut-offs: UIC < 150, 150–249, 250–499 and ≥500 reflecting insufficient, adequate, above requirements and excessive iodine intakes, respectively.

**Setting:**

Mangochi District, Malawi.

**Participants:**

Women ≤20 weeks pregnant.

**Results:**

Groups had comparable background characteristics. At baseline, overall median (Q1, Q3) UIC (319 (167, 559)) suggested iodine intakes above requirements. At 36 weeks, the geometric mean (95 % CI) UIC of the IFA (197 (171, 226)), MMN (212 (185, 243)) and LNS (220 (192, 253)) groups did not differ (*P* = 0·53) and reflected adequate intakes.

**Conclusions:**

In this setting, provision of supplements containing iodine at the recommended dose to pregnant women with relatively high iodine intakes at baseline, presumably from iodised salt, has no impact on the women’s UIC. Regular monitoring of the iodine status of pregnant women in such settings is advisable. Clinicaltrials.gov identifier: NCT01239693.

Micronutrient deficiencies are common among pregnant women in many low-income settings^(1,2)^. For iodine deficiency, even a mild form during pregnancy could have serious consequences^(3,4)^, due to the mineral’s essential role in the synthesis of thyroid hormones, which are required for normal body metabolism and the development of the central nervous system in fetuses and children^(5)^. In 2012, >40 % of the population of Africa were estimated to have insufficient iodine intake^(6)^. For pregnant women, no such estimate is currently available^(6,7)^, but iodine inadequacy may be common because of increased iodine needs during pregnancy^(8)^ and the possibility that available salt may not be adequately iodised^(9)^ even when a salt iodisation programme exists. Iodine deficiency is associated with miscarriage, brain damage and delivery of still-born, preterm or neuro-cognitively impaired infants^(5)^ and is identified as a key risk factor preventing children from achieving their developmental potential^(10)^.

Investigators participating in the International Lipid-based Nutrient Supplements (iLiNS) Project developed small-quantity lipid-based nutrient supplements (SQ-LNS) for enriching home-prepared foods for pregnant and lactating women, to increase intakes of micronutrients and essential fatty acids^(11)^. A daily dose of the SQ-LNS contains the WHO-recommended amount of iodine for pregnancy^(12)^. We evaluated the efficacy of the SQ-LNS among pregnant women in a semi-urban setting in Ghana^(13)^ and a predominantly rural site in Malawi^(14)^. In both countries, micronutrient deficiencies are common and there is a national salt iodisation programme, but both countries face various challenges in the implementation of the programme, including the presence of small-scale salt producers with little or no capacity to iodise the salt they produce, poor packaging and handling of iodised salt resulting in excess loss of added iodine^(15–17)^ and resource constraints hampering monitoring and quality assurance^(18)^.

Few trials comparing the impact of multiple micronutrient supplementation *v*. Fe and folic acid supplementation during pregnancy^(19)^ have reported on women’s urinary iodine concentration (UIC), despite the international efforts at eliminating iodine deficiency^(18)^. In the Ghana trial^(20)^, the iodine intakes of the pregnant women at baseline were within the WHO ‘insufficient’ range despite the national salt iodisation programme, and the provision of SQ-LNS (or multiple micronutrients (MMN) with similar iodine content as the SQ-LNS) increased the likelihood of adequate iodine status at 36 weeks of gestation. In the present pre-specified secondary outcome analysis, we aimed to compare the UIC among the three groups of pregnant women enroled in the Malawi trial^(14)^ and to assess the groups’ iodine intake adequacy using WHO cut-offs.

## Methods

### Study design, site and participants

The design, site and participants of the iLiNS DYAD-Malawi trial have been described previously^(14)^. In brief, this was a partially double-blind, individually randomised, controlled trial with three equal-size intervention groups conducted in the Mangochi District, south of Malawi. This area is largely rural, with the main source of livelihood being subsistence farming and fishing. The diet of people in the area consists predominantly of cereals mainly maize, with relatively small amounts of roots, tubers, fish, fruit and legumes^(21)^.

The trial participants were pregnant women presenting at antenatal clinics at four health facilities in the area between February 2011 and August 2012, including the district hospital in Mangochi, a semi-private hospital at Malindi and two public health centres at Lungwena and Namwera. Women were eligible if all of the following applied: no more than 20 completed weeks of pregnancy confirmed by an ultrasound scan; residence in the catchment area served by the four health facilities; no intention to travel out of the study area during the period of the intervention and signed or thumb-printed informed consent. Women were excluded if any of the following was present: (1) < 15 years of age; (2) requiring medical attention due to a chronic health condition; (3) known to be asthmatic or allergic to peanuts or to any substance; (4) severe illness warranting hospital referral or emergency medical care; (5) history of anaphylaxis; (6) pregnancy complications at the time of enrolment, including moderate to severe oedema, blood Hb concentration < 50 g/L, systolic blood pressure (BP) > 160 mmHg or diastolic BP > 100 mmHg; (7) history of being enroled in the iLiNS DYAD-Malawi trial during a previous pregnancy or (8) currently taking part in another clinical trial.

### Intervention groups and study supplements

The intervention groups were (1) the iron–folic acid group (hereinafter, IFA) assigned to the standard nutritional supplementation for pregnant women in Malawi consisting of 60 mg/d Fe and 400 μg/d folic acid; (2) the MMN group assigned to 18 vitamins and minerals (including 20 mg/d Fe) and (3) the lipid-based nutrient supplements group (LNS) assigned to 20 g/d SQ-LNS containing similar micronutrients as the MMN group, and in addition, energy (494 kJ/d or 118 kcal/d), protein, essential fatty acids and the maximum amounts of Ca, Mg, P and K that could be included given technical and organoleptic constraints^(11)^. The IFA and MMN groups served as controls.

The nutrient contents of the supplements used in the trial^(14)^ are presented in Table 1, and the rationale for the nutrient contents and concentrations of those for the MMN and LNS groups were reported previously^(11)^. The supplements for the IFA and MMN groups were supplied by DSM Nutritional Products (Pty) Ltd in 10-capsule blister packs, while the SQ-LNS for the LNS group were produced by Nutriset S.A.S. (Malaunay, France) in individual 20 g sachets. Most of the vitamins and minerals in the MMN and SQ-LNS supplements were included at a dose of either *1x* or *2x* the RDA for pregnancy^(11)^. We included 250 µg/d for iodine based on the WHO recommendation^(12)^.

**Table 1:** Composition of the supplements used in the iLiNS DYAD micronutrient supplementation trial in rural Malawi, by group

Nutrient	IFA	MMN	SQ-LNS
Ration/day	1 tablet	1 tablet	20 g
Total energy (kJ)	0	0	494
Protein (g)	0	0	2·6
Fat (g)	0	0	10
Linoleic acid (g)	0	0	4·59
*α*-Linolenic acid (g)	0	0	0·59
Vitamin A (μg RE)	0	800	800
Vitamin C (mg)	0	100	100
Vitamin B_1_(mg)	0	2·8	2·8
Vitamin B_2_ (mg)	0	2·8	2·8
Niacin (mg)	0	36	36
Folic acid (μg)	400	400	400
Pantothenic acid (mg)	0	7	7
Vitamin B_6_ (mg)	0	3·8	3·8
Vitamin B_12_ (μg)	0	5·2	5·2
Vitamin D (IU)	0	400	400
Vitamin E (mg)	0	20	20
Vitamin K (μg)	0	45	45
Fe (mg)	60	20	20
Zn (mg)	0	30	30
Cu (mg)	0	4	4
Ca (mg)	0	0	280
P (mg)	0	0	190
K (mg)	0	0	200
Mg (mg)	0	0	65
Se (μg)	0	130	130
Iodine (μg)	0	250	250
Mn (mg)	0	2·6	2·6

IFA, iron and folic acid: MMN, multiple micronutrient supplement; SQ-LNS, small-quantity lipid-based nutrient supplement. IFA was standard nutritional supplementation at the time of the study. Nutrient concentrations for SQ-LNS include contributions from the ingredients and from the multiple micronutrient premix. Total energy is equivalent to 118 kcal.

All supplements were intended to be consumed daily: for the IFA and MMN groups, 1 capsule was to be taken with water after a meal, and for the LNS group, 1 sachet of SQ-LNS was to be mixed with a small quantity of any food and consumed during the day. At the project office, supplements were stored at room temperature (20 °C–40 °C) in cardboard boxes away from light. In the homes, women were advised to store the supplement in dry places indoors.

### Randomisation and enrolment

Pregnant women were considered enroled into the trial if they participated in a baseline assessment and were subsequently randomised into one of the three intervention groups. As previously described^(14)^, the randomisation of the women was completed as follows: first, the Study Statistician at UC Davis created four randomisation code lists in blocks of nine, one list for each of the four health facilities serving as enrolment sites. To maintain blinding, each supplement or intervention group was coded using three different alphabetical letters between ‘A’ and ‘M’, so that field workers could identify women only by the alphabetical letters. Next, a researcher independent of the trial created randomisation slips for each enrolment site; each slip contained the letter code indicating the group assignment and a unique identification number for the participant. These slips were placed in opaque envelopes, which were sealed, labelled with the block numbers and individual enrolment numbers and stacked in increasing order of the block numbers. In order to not influence the women’s choices of envelopes when asked to make a pick from among a set of envelopes, we tried to hide the labels by writing in pencil on one side of the envelope that was never shown to the women.

At each enrolment, the randomiser shuffled the six topmost envelopes in the stack and asked the potential participant to choose one, which revealed the group assignment and identification number. The randomiser then returned the unused envelopes to the top of the stack. The process was repeated until all of the envelopes prepared for the enrolment site were used. When there were less than six envelopes left for a participant to pick from, the randomiser presented whatever number of envelopes that remained. At no point during randomisation, however, was it possible for the randomiser to guess the remaining allocations, since he or she had no knowledge of the randomisation scheme.

After randomisation, women received a 2-week supply of the assigned supplement along with the instructions on how to consume it. We told women not to consume more than the recommended dose each day even if they missed taking the supplement the previous day or days.

### Data collection and follow-up procedures

During the baseline assessment, trained anthropometrists measured women’s weight (SECA 874), height (Harpenden stadiometer) and mid-upper arm circumference (Shorrtape, Weigh and Measure, LLC). Research nurses determined women’s malaria parasitaemia by rapid diagnostics tests (Clearview Malaria Combo), Hb concentration by Hemocue (HemoCue AB) and gestational age by fetal dimensions (biparietal diameter, femur length and abdominal circumference) and ultrasound examination (EDAN DUS 3 Digital Ultrasonic Diagnostic Imaging System, EDAN Instruments, Inc.). In addition, the research nurses obtained spot urine samples from women, which were frozen until analysed. Finally, study workers collected women’s background demographic and socio-economic information at enrolment.

Following enrolment, field workers visited women in the homes every 2 weeks, whereupon they delivered fresh supplies of supplements and monitored adherence by counting the numbers of delivered and recovered capsules or sachets. As reported previously^(14)^, we suspended the distribution supplements to women in the LNS group for a period from 1 through 21 August 2012, on the advice of the trial’s DSMB due to a new recommendation involving the testing of LNS products for the presence of *Cronobacter sakazakii.* During this period, 160 pregnant women in the LNS group who missed their supplements for a number of days ranging from 1 to 20 were provided with Fe and folic acid capsules (as were women in the IFA group), in accordance with standard guidelines in Malawi. However, a total of 33 women who missed receiving their assigned supplement could not be reached during the temporary Fe and folic acid distribution, since they were not available at their homes during the time. We resumed the distribution of the intended supplements to the LNS group after 22 August 2012. At a laboratory visit at 36 weeks of gestation, study nurses collected spot urine samples as done previously.

Because of the apparent differences between the capsules (IFA and MMN) and the SQ-LNS, it was not possible to blind field workers and participants to women receiving these supplements. The study staff who collected or analysed the samples were, however, blinded to the group assignments.

#### Determination of urinary iodine concentration

We air freighted the urine samples on dry ice to the laboratories of the Medical Research Council in Cape Town, South Africa, where UIC was determined. The urine samples were manually digested in a 96-well plate by using ammonium persulphate. The digested samples were then transferred to a new microplate for the Sandell–Kolthoff reaction, and UIC was read at 405 nm^(22,23)^. Urine samples collected at enrolment and at 36 weeks of gestation were analysed at the same time.

In the present analysis, the secondary outcomes evaluated were geometric mean UIC (µg/L) at 36 weeks of gestation and median change in UIC from enrolment to 36 weeks of gestation.

### Sample size calculation and data analysis

As with many continuous biochemical outcomes in the trial, the target sample size for women’s UIC at 36 weeks of pregnancy was based on detecting an effect size or Cohen’s *d* ^(24)^ of ≥0·5 between any two groups, with a two-sided 5 % test and 80 % power. Thus, approximately 105 women per group (or 315 women for the 3 groups) were required, after taking into account up to 25 % attrition. To reduce the risk of missing data, the sub-sample for the UIC analysis was selected from among women for whom urine samples collected at baseline and at 36 weeks of gestation were both available. At 36 weeks of gestation, we had UIC values for 313 women in the three groups, which gave > 90 % power to detect an effect size (Cohen’s *d*) of 0·5 between any two groups.

Before we began the present analysis, we prepared and posted the statistical analysis plan at our website (www.ilins.org). In both the statistical analysis plan and the trial protocol described at ClinicalTrials.org, we listed women’s UIC as a secondary outcome intended to be analysed separately. We performed all analyses using SAS for Windows Release 9.4 and included women in the analysis as randomised, regardless of adherence to treatment.

We calculated the household assets index, housing index and Household Food Insecurity Access Scale score as proxy indicators of background socio-economic status, by using principal component analysis^(25)^. We summarised the background characteristics at enrolment, by group, as mean ± sd or frequency (%). Because UIC are known to be not normally distributed^(26)^, we summarised UIC at baseline and 36 weeks of gestation and the change in UIC from baseline to 36 weeks as median and first and third quartiles (Q1, Q3).

We assessed the impact of the intervention on women’s UIC at 36 weeks of gestation by comparing the three groups using ANCOVA (SAS PROC GLIMMIX) with Tukey adjustment for multiple comparison, after natural log transforming all UIC values^(20)^. We performed the ANCOVA twice^(20)^: in the first, we included log-transformed baseline UIC as the only pre-specified covariate in the model; in the second, we included additional pre-specified background covariates in the model, if they were significantly associated with the outcome (log-transformed UIC at 36 weeks of gestation) at ≤ 0·1 level of significance. Pre-specified background variables evaluated as potential covariates in bivariate models were gestational age at enrolment, years of formal education, parity (nulliparous or parous), household assets index, Household Food Insecurity Access Scale^(25)^ and season of enrolment (wet or not wet). From the ANCOVA models, we calculated the adjusted group geometric mean UIC at 36 weeks of gestation and their 95 % CI by back transformation. The geometric mean UIC is considered an approximate estimator of median UIC^(27,28)^. We examined whether there were significant interaction effects (*P*_for interaction_ < 0·1) between treatment and the pre-specified background variables on women’s UIC at 36 weeks of gestation.

To assess the adequacy of the women’s iodine intakes using WHO guidelines, we compared the group median UIC at baseline and the group median as well as geometric mean UIC at 36 weeks (approximately the median of the untransformed values) with WHO cut-offs, whereby median UIC <150, 150–249, 250–499 and ≥500 µg/L represent ‘inadequate’, ‘adequate’, ‘above requirements’ and ‘excessive – i.e. in excess of the amount required to prevent and control iodine deficiency’ iodine intakes, respectively^(26)^. Median UIC from spot urine samples^(29)^ and the cut-offs^(26)^ are recommended for assessing iodine intakes in populations^(26)^.

Statistics in the texts are median (Q1, Q3) or geometric mean (95 % CI). As previously reported^(30)^, women’s adherence to supplement intake during pregnancy (i.e. median percentage of follow-up days women self-reported consuming the supplements) was 91·7 % for the IFA group; 91·0 % for the MMN group and 93·8 % for the LNS group^(30)^.

## Results

A total of 1391 pregnant women were enroled into the iLiNS DYAD-Malawi trial. In Table 2, we show the background characteristics of the 317 women whose spot UIC were analysed herein, by group. These characteristics were comparable across the three groups. For example, on average, the women were in their mid-twenties, had <4 y of formal education and had mean household assets index and housing quality scores that suggested generally low socio-economic status. Women were enroled at a mean gestational age of 16·8 weeks, ~21 % of them had not had children before, ~20 % were anaemic and ~23 % tested positive in the rapid diagnostic test for malaria. Between women in the UIC analysis sub-sample and those (*n* 1073) not selected for UIC analysis, there were no significant differences in the background characteristics listed in Table 2 (full results not shown), except that the former had lower mean ± sd years of formal education (3·5 ± 3·4 [*n* 314] *v*. 4·2 ± 3·5 [1011]; *P* = 0·006) and lower mean ± sd housing quality score (−0·15 ± 0·84 [315] *v*. 0·05 ± 1·04 [1001]; *P* = 0·002) suggesting that those women generally had a lower socio-economic status.

**Table 2:** Background characteristics at enrolment of women randomly selected for urinary iodine analysis among pregnant women enroled in the iLiNS DYAD micronutrient supplementation trial in rural Malawi, by group

	IFA (*n* 104)				MMN (*n* 106)				LNS (*n* 107)			
Characteristics	*n*	%	Mean	sd	*n*	%	Mean	sd	*n*	%	Mean	sd
Age, y	104	–	25	6	106	–	25	6	107	–	26	7
Years of formal education, y	103	–	4	3	105	–	4	3	106	–	4	4
Weight at enrolment, kg	104	–	54·6	7·6	105	–	53·2	7·8	107	–	54·1	9·1
Height at enrolment, cm	104	–	156	6	105	–	156	6	106	–	156	6
BMI at enrolment, kg/m^2^	104	–	22·3	2·4	105	–	22	2·9	106	–	22·1	3·4
Household assets index^*^	104	–	−0·08	0·85	102	–	−0·04	0·80	106	–	−0·14	0·74
Housing quality score^*^	104	–	−0·26	0·82	106	–	−0·13	0·84	105	–	−0·08	0·85
HFIAS score^†^	104	–	5·2	3·8	103	–	5·8	4·5	106	–	4·7	4·2
Gestational age at enrolment, week	104	–	16·6	2·2	106	–	16·8	2·2	107	–	16·9	2·2
Blood Hg concentration, g/L	104	–	111	16	105	–	112	13·5	107	–	111	15·8
Season of enrolment = Rainy Season	104	57·7	–	–	106	59·4	–	–	107	57·9	–	–
Nulliparous,	104	20·2	–	–	105	22·9	–	–	107	21·5	–	–
Malarial RDT^‡^	104	23·1	–	–	105	22·9	–	–	105	24·8	–	–
Anaemia^§^	104	17·3	–	–	105	20	–	–	107	24·3	–	–

IFA, iron and folic acid: MMN, multiple micronutrient supplement; SQ-LNS, small-quantity lipid-based nutrient; HFIAS, Household Food Insecurity Access Scale; RDT, Rapid Diagnostic Test. IFA group was assigned to consume 60 mg/d Fe and 400 µg/d folic acid; MMN group was assigned to consume 18 vitamins and minerals (including 20 mg/d Fe and 250 µg/d iodine); LNS group was assigned to consume 20 g/d SQ-LNS with the same micronutrients as the MMN group plus Ca, P, K and Mg and macronutrients. Total *n* 317.

* Proxy indices for household socioeconomic status obtained using principal component analysis; higher values represent higher socioeconomic status.

† HFIAS, Household Food Insecurity Access Scale is a proxy indicator for household food insecurity^(25)^; higher values represent higher food insecurity.

‡ RDT (Clearview Malarial Combo, Vision Biotech, South Africa) detected *P. falciparum* and non-*P. falciparum* histidine-rich protein-2.

§ Anaemia defined as blood Hg concentration <100 g/L^(53)^.

Results of the analysis of the UIC of women, by group at baseline and 36 weeks of gestation, are presented in Table 3. At baseline, the three groups appeared to have similar median UIC, with an overall median (Q1, Q3) UIC of 319 (167, 559) µg/L. At that point, the median UIC of the women, regardless of group allocation, was within the WHO cut-off range of 250–499 µg/L indicating iodine intakes ‘above requirements’. The median change in UIC from baseline to 36 weeks of gestation was negative for all three groups (signifying a decrease in UIC during the period), with the overall median change being −84 (−350, 71) µg/L.

**Table 3:** Urinary iodine concentration (µg/L) of pregnant women who participated in the iLiNS DYAD micronutrient supplementation trial in rural Malawi, by group

	IFA (*n* 104)				MMN (*n* 106)				LNS (*n* 107)				
Urinary iodine concentration, µg/L	*n*	Median or geometric mean	IQR or 95 % CI		*n*	Median or geometric mean	IQR or 95 % CI		*n*	Median or geometric mean	IQR or 95 % CI		*P*
Baseline*	103	264	147,	553	106	300	159,	535	105	352	229,	619	–
36 weeks of gestation*	102	183	106,	328	105	194	128,	328	106	207	127,	373	–
Change (enrolment to 36 weeks of gestation)*	101	−69	−344,	87	106	−88	−343,	86	104	−97	−362,	63	–
36 weeks of gestation†	102	197	171,	226	105	212	185,	243	106	220	192,	253	0·53
36 weeks of gestation‡	100	197	171,	226	99	212	185,	244	102	217	189,	249	0·60

IFA, iron and folic acid: MMN, multiple micronutrient supplement; SQ-LNS, small-quantity lipid-based nutrient; IQR, interquartile range. IFA group was assigned to consume 60 mg/d Fe and 400 µg/d folic acid; MMN group was assigned to consume 18 vitamins and minerals (including 20 mg/d Fe and 250 µg/d iodine); LNS group was assigned to consume 20 g/d SQ-LNS with the same micronutrients as the MMN group plus Ca, P, K and Mg and macronutrients. Total *n* 317.

* Values are *n*, median and IQR.

† Values at 36 weeks of gestation are *n*, geometric mean and 95 % CI. The geometric mean and 95 % CI were based on ANCOVA (SAS PROC GLIMMIX) with Tukey adjustment for multiple comparison, after controlling for baseline urinary iodine concentration (UIC) and back-transforming the log-mean UIC.

‡ Values at 36 weeks of gestation are *n*, geometric mean and 95 % CI. The geometric mean and 95 % CI were based on ANCOVA (SAS PROC GLIMMIX) with Tukey adjustment for multiple comparison, after controlling for baseline urinary iodine concentration (UIC) as well as household assets index (proxy indicator for household socioeconomic status obtained using principal component analysis), years of formal education, and parity (nulliparous or parous), and back-transforming the log-mean UIC.

At 36 weeks of gestation, the IFA group consuming the supplement (Fe and folic acid) with no added iodine had a median UIC (183 µg/L) that was within the WHO cut-off range (150–249 µg/L) for ‘adequate intakes’. Median UIC suggestive of adequate iodine intakes at 36 weeks were observed for both the MMN and LNS groups in which women consumed supplements with added iodine. In the ANCOVA model controlling for baseline UIC only, the geometric mean (95 % CI) UIC of the three groups did not differ significantly (*P* = 0·53), and these findings remained unchanged when controlling for additional background characteristics including household assets index, years of formal education and parity, which were associated with UIC at 36 weeks of gestation at ≤0·1 level of significance in bivariate analyses. As was the case for the untransformed median values, the geometric mean UIC of each group was within the WHO cut-off range (150–249 µg/L) for ‘adequate intakes’. In the analysis examining potential effect modification, we found no significant interactions between intervention group and gestational age at enrolment, years of formal education, parity, household assets index, Household Food Insecurity Access Scale or season of enrolment (wet or not wet).

## Discussion

In this rural Malawi setting, the provision of SQ-LNS or multiple micronutrient supplements (each containing the 250 µg/d WHO-recommended daily dose of iodine) starting before 20 wk gestation did not affect the women’s geometric mean UIC at 36 weeks of pregnancy, compared with the provision of Fe and folic acid. At enrolment, the women’s median UIC suggested that their average iodine intakes were above the WHO requirement for preventing and controlling iodine deficiency; at 36 weeks of pregnancy, across all three intervention groups, the geometric mean UIC was within the WHO range for ‘adequate’ intakes.

UIC is one of four indicators, along with thyroid size, serum thyroid-stimulating hormone (TSH) and serum thyroglobulin (Tg), generally recommended by the WHO for assessing the impact of interventions on the iodine status of populations^(29)^; serum thyroxin (T4) and triiodothyronine (T3) are not usually recommended because these tests are more cumbersome and expensive and yet less sensitive^(29,31)^. We used spot UIC in our study because it is widely accepted and is relatively easy and inexpensive to measure, although it is not useful for estimating iodine intakes in individuals^(26,31,32)^ and provides no direct information about thyroid function^(12,26)^. While urinary iodine excretion over 24 h is appropriate for estimating the iodine intakes of individuals, the process is cumbersome and may be incomplete^(26,32,33)^. We did not measure thyroid size because Malawi had an ongoing universal salt iodisation programme, and therefore, this indicator may not have been useful^(29)^. Of the two blood constituents, TSH is not a sensitive indicator for adults and so was not a good choice for our study population^(29,31)^. However, serum Tg might have been useful because it is appropriate for pregnant women^(34,35)^, sensitive to iodine intakes over a period of months (or years) and has an established international reference range^(29,31)^, although it is not always reliable for pregnant women^(32)^.

Results regarding spot UIC from similar micronutrient supplementation trials for pregnant women were reported previously from Ghana and Bangladesh. In the Ghana trial^(20)^, which had the same design as this trial in Malawi, women who received SQ-LNS or MMN (containing similar nutrient contents as those used in this trial) from ≤ 20 weeks of pregnancy had greater geometric mean UIC than their counterparts who received Fe and folic acid. The Bangladesh study^(36)^ was a cluster-randomised trial in which women received either SQ-LNS with the same nutrient contents as those provided in Ghana^(20)^ and Malawi, or 60 mg/d Fe and 400 µg/d folic acid from ≤ 20 weeks of pregnancy. At 36 weeks of pregnancy, the two groups did not differ significantly in geometric mean UIC. Investigators speculated that the lack of impact of SQ-LNS consumption in the Bangladesh trial^(36)^ on UIC was due to the very low baseline median UIC (46–50 µg/L) in that setting (compared with 119–151 µg/L in Ghana^(20)^), which may have resulted in the supplemental iodine being taken up by maternal and possibly fetal iodine-deprived thyroid glands for thyroid hormones production, rather than being excreted in the women’s urine.

The reason for no differences in geometric mean UIC at 36 weeks of pregnancy between women receiving supplements containing the WHO-recommended daily iodine dose *v*. those in the IFA group in the present study in Malawi is not entirely clear, given that a positive impact of the iodine supplementation was observed in Ghana^(20)^, and the mean percentage of days in the study that MMN and SQ-LNS supplements were self-reportedly consumed was greater in Malawi than in Ghana for the MMN (89·2 % *v*. 81·9 %; *P* < 0·001) and LNS (90·6 % *v*. 78·2 %; *P* < 0·001) groups^(30)^. It is possible that mineral interactions (e.g. involving iodine and Fe) may have reduced the bioavailability of potassium iodate in the SQ-LNS and multiple micronutrient supplements^(37)^, but this is unlikely because the same supplements were used in Ghana^(20)^. It is also unlikely that the Malawian pregnant women were unable to absorb iodine due to factors such as environmental enteropathy^(38)^, given that the women’s baseline median UIC was high compared with that observed in Ghana^(20)^ and Bangladesh^(36)^.

Rather, the reason for the lack of impact on women’s UIC in Malawi may be related to the relatively high median UIC (which reflected relatively high iodine intakes) at baseline. It is conceivable that the consumption of the iodine-containing supplements resulted in prolonged, modestly excessive iodine intakes. Under this circumstance, one adaptation may be the so-called ‘escape’ from the Wolff-Chaikoff effect^(39)^, in which the sodium-iodide symporter glycoprotein that mediates the active transport of iodide in the small intestines^(40,41)^ as well as the thyroid, the placental-fetal system^(42–44)^ and other tissues^(44,45)^, may be down-regulated^(40,46,47)^. This may have reduced iodide absorption in the small intestine^(40,41)^ and also transport into the thyroid^(44,46)^ and subsequently prevented an increase in the UIC of women in the MMN and LNS groups. It is also possible that a thyroid-gastric system of controlling circulating levels of iodine, by which absorbed iodine may be secreted out of circulation back into the gastrointestinal tract and lost through feces^(45)^, may have played a role in preventing an increase in the UIC of women in the MMN and LNS groups.

The median UIC values observed for the Malawian pregnant women in our study were generally similar to values in other recent reports^(17,48)^. Among 118 female non-pregnant volunteers 18–50 years of age from six villages in northern Malawi and six villages in southern Malawi^(17)^, median (Q1, Q3) UIC was reported to be 222 (141, 344) μg/L. The Malawi Micronutrient Survey 2015–2016 included a nationally representative sample of non-pregnant women 15–49 years of age^(48)^, and the reported median (Q1, Q3) UIC was 271 (158, 384). In our study, the median UIC of the pregnant women at enrolment was slightly higher than reported in these two studies^(17,48)^, perhaps because urinary iodine excretion tends to increase during pregnancy^(43)^. At 36 weeks of pregnancy, the group geometric mean UIC in our study were slightly lower than the median value of the non-pregnant women in the Malawi Micronutrient Survey 2015–2016^(48)^, likely because of the increased demand for iodine imposed by pregnancy^(26)^.

We speculate that the use of iodised salt is one of the drivers behind the apparent relatively high iodine intakes of the women in our study, based on several considerations. First, in one report in which soil (*n* 92) and drinking water (hand-dug wells and boreholes, *n* 19) samples from across Malawi^(17)^ were analysed, the median iodine concentrations were 2·06 mg/kg for soil and 12·6 μg/L for water. These values appear relatively low compared with those observed elsewhere in Africa such as Morocco, where average soil and water iodine concentrations of 2·76 mg/kg and 17·8 μg/L, respectively, were found in one area^(49)^. Second, in another study of villages selected from northern and southern Malawi^(17)^, the median iodine concentrations of staple foods were estimated to be 10·0 μg/kg for maize, 8·0 μg/kg for roots and tubers, 155·0 μg/kg for green leafy vegetables and 510·0 μg/kg for freshwater fish, which are lower than the averages of the values (μg/kg) reported in the literature for maize (80·0) and leafy vegetables (171·0), though not for freshwater fish (101·5)^(50)^. Third, in an analysis using data from the FAO Food Balance Sheet for 2011^(17)^, the Malawian national per capita iodine supply from foods other than salt was estimated to be only 7·8 μg/d, confirming that the iodine supply from the diet tends to be low.

Malawi was credited in 2013 as having achieved a household coverage of > 90 % for iodised salt^(51)^. More recently, the Malawi Micronutrients Survey 2015–2016^(48)^ showed that among all households, at least 75 % had iodised salt: 41 % had salt with ‘adequate’ (15–39·9 ppm) iodine, 34 % had salt with ‘excess’ (≥ 40 ppm) iodine and 25 % had salt with ‘inadequate’ or no measurable iodine (<15 ppm). We did not collect data on iodised salt consumption in our study, but information from the Malawi Micronutrients Survey 2015–2016^(48)^ suggests that the use of iodised salt could be responsible for the relatively high UIC in our sample. In fact, given our finding that the women’s UIC at enrolment was within the WHO cut-off range for ‘above requirements’, consumption of salt with excess iodine concentration may have been common. At least two other previous reports^(16,17)^ showed that the iodine content of salt sold in some outlets in Malawi was too high. Persistent intakes of iodine higher than needed may impair thyroid function by inhibiting the synthesis and release of thyroid hormones^(52)^.

The strengths of our study included (a) randomisation of treatment groups, (b) blinding of the outcome assessors to group assignments, (c) intense follow-up of participants and (d) rigorous quality assurance in data collection. A key weakness is that we measured only UIC and no other biomarkers of iodine status, such as serum Tg. For this reason, we do not know whether women in the MMN and LNS groups differed in their thyroid function, possibly reflecting higher iodine intakes, when compared with those in the IFA group.

We conclude that in this rural Malawi setting, the provision of supplements containing the current WHO-recommended daily dose of iodine, compared with Fe and folic acid, has no impact on the women’s UIC at 36 weeks of gestation, probably because baseline iodine intakes were more than adequate. Regular monitoring of the iodine status of pregnant women in this setting may be useful to prevent excessive intakes of iodine.
